# Broadband Linear High-Power Amplifier Based on the Parallel Amplification Architecture for Electromagnetic Ultrasonic Guided Wave

**DOI:** 10.3390/s19132924

**Published:** 2019-07-02

**Authors:** Jinjie Zhou, Yang Zheng

**Affiliations:** 1 School of Mechanical Engineering, North University of China, Taiyuan 030051, China; 2 China Special Equipment Inspection and Research Institute, Beijing 100029, China

**Keywords:** linear power amplifier, high-output power, electromagnetic acoustic transducer (EMAT), ultrasonic guided wave (UGW)

## Abstract

The linear power amplifier with high-output power in the broadband frequency is the critical component required by exciting the electromagnetic acoustic transducer (EMAT) to generate ultrasonic guided wave (UGW). The methods to realize the output of a high-power signal in the linear amplification mode and to expand the bandwidth at high-output power are seldom reported. To solve these problems, a power amplifier with differential structure is developed by using the parallel amplification architecture and the broadband feedback circuits. The proposed power amplifier uses a differential structure to suppress the even harmonic waves and remove the disruptions. Each branch of the differential structure consists of five linear power amplifier modules with output terminals connected in parallel to increase the output power. Also, the negative voltage feedback is used to extend the bandwidth of the power amplifier. The experimental results show that the −3 dB bandwidth of the amplifier is from 40 kHz to 2.5 MHz, and the transient output power is greater than 1 kW. The power amplifier can drive the EMATs to generate ultrasonic guided waves. Because of the high-output power and good linearity, the proposed power amplifier has excellent potential for EMAT UGW applications.

## 1. Introduction

Ultrasonic guided waves can be excited at one point and propagate a very long distance along wave guide structures, such as pipe-like or plate-like structures [1,2]. In addition, the sound field of the ultrasonic guided waves can cover the entire range of the wall thickness. Thus, ultrasonic guided waves are especially suitable for the large-scale NDT and NDE applications of wave guide structures. Compared with piezoelectric transducers, the contactless characteristic of EMATs overcomes the need of couplant between the conductive materials and the transducer itself [3,4,5] and makes them particularly suitable for NDT and NDE at high temperatures [6,7,8]. Furthermore, EMATs can generate a single wave mode, such as S0 or SH mode, by varying the shape and array of magnets and coils, which is very difficult to generate with piezoelectric transducers [2,3,4]. Electromagnetic ultrasonic guided wave techniques integrate the advantages of EMATs and guided waves. This technology has been demonstrated to offer great promise for the in-line inspection of pipelines [5], high-temperature plates testing of the solar thermal industry [7], and so on.

In the development of the EMAT inspection system for guided waves, power amplifier design is one of the most critical and challenging works. The power amplifier is employed to amplify the small signal from the analog signal source and drives the EMAT to generate ultrasonic guided waves. The characteristics of the power amplifier affect the generation of ultrasound guided waves. Commonly, two main objects must be simultaneously achieved for this kind of power amplifier design: (1) The amplifier should be able to work in the linear amplification mode across a range of frequencies. The frequency range of the exciting signal to generate the UGWs is often 50–500 kHz [1,2,3,9]. Because of the multimode phenomenon and dispersion effect, the guided wave signals become difficult to analyze [10]. The multimode phenomenon can be resolved by the special design of transducers. However, to suppress the dispersion effect of the guided waves, the short-time and narrow-band pulses, such as rectangular [1], Hanning [2,9], and Gaussian [3] windowed sine signals at the appreciated operating frequency are used as exciting signals. The short-time and narrow-band pulses are very suitable for amplification by using a linear amplification method. (2) One should output high-power exciting signals into an inductive load to drive the EMAT. The main reasons are that the energy conversion efficiency of EMATs is extremely low [11], and peak powers greater than 1 kW are required to excite an EMAT [12].

In the past few decades, many pulses or switch-mode power amplifiers (PAs) or linear PAs were designed for exciting ultrasonic signals. Because the ultrasonic signal is nondispersion, the short-time signal with both broad and narrow bands can be used as an exciting signal. Many high-voltage pulses were designed for exciting ultrasonic signals with piezoelectric transducers [13,14,15,16,17,18,19,20] and EMATs [21]. These pulses can produce unipolar or bipolar square wave signals, and the square wave may have a certain tilt in the high half cycle or the low half cycle. However, they cannot produce the short-time and narrow-band signals for ultrasonic guided wave detection.

Both high-output power and high efficiency can be realized by switch-mode PAs, but the output matching network only generates high-power signals with low frequencies of 10–100 kHz [22] as the output. Because the windowed modulation sinusoidal signals vary more severely than those without modulation, a pulse-width modulation signal should make the power MOSFETs work at least twenty times higher than the input signal center frequency. To amplify a 500 kHz windowed modulation sinusoidal signal, the switching frequency is at least 10 MHz. At this frequency, it is difficult to design a high-speed square-wave driving circuit. Most power MOSFETs cannot rise from the closed state to the high-voltage state within a very short time.

In principle, to amplify a hundreds of kHz signal to a high voltage is not a problem if one uses linear PAs. Linear PAs often use Class AB configuration whose two branches are respectively driven by differential signals and outputted with the transformer. The static operating point of each branch with the same N-channel MOSFETs linear amplifier circuit is slightly higher than the open voltage. Class AB configuration solves the contradiction between improving efficiency and reducing waveform distortion, so it is often used to amplify the signal from hundreds of kHz [23] to tens of MHz [24,25] to a high-voltage signal for the high-impedance transducer. However, these linear PAs were designed to excite piezoelectric transducers, and their output powers are too small to excite EMATs.

The method to achieve the output of a high-power signal in the linear amplification mode was not reported in detail. The problem that follows is how to expand the bandwidth at a high-power output. To add channels to construct an array detection system, a customized linear PA to excite EMATs to generate UGWs is very desirable. The current publication aims to study the linear amplification method of a kilowatt high-output-power signal for the EMAT. Multiple power MOSFETs with linear amplification modules (LAMs) operating in parallel are used to improve the output power of the designed linear PA. The negative voltage feedback is used to extend the bandwidth of the linear PA. Therefore, a linear PA is developed, which can achieve an output power that is always more than 1 kW at the −3 dB bandwidth from 40 kHz to 2.5 MHz. The parameters of the proposed PA are tested, and its performance is verified by exciting the EMAT to generate UGW.

## 2. Configuration Selection

### 2.1. Performance Evaluation of the Parallel LAMs

Due to the limitation of fabrication technology and the heat dissipation conditions of power devices, it is difficult for the Class AB linear PA with two power MOSFETs to output thousands of watts. The challenges of expanding the transient outputting power of a PA that functions in a linear amplification mode must be carefully considered. The output stage is the most key model of linear PA, and its performance decides the maximum output power. At radio frequency and Ku-band, the linear PA can reach the required high power by using a power combiner to combine the output of each small-power amplifier module [26,27,28,29,30]. The ultrasound driver that sends square-wave signals uses eight [31] or fourteen [32] MOSFETs in parallel to increase the output power. Therefore, it is worth considering that the output power can be increased by using multiple LAMs in parallel.

**Figure 1:**
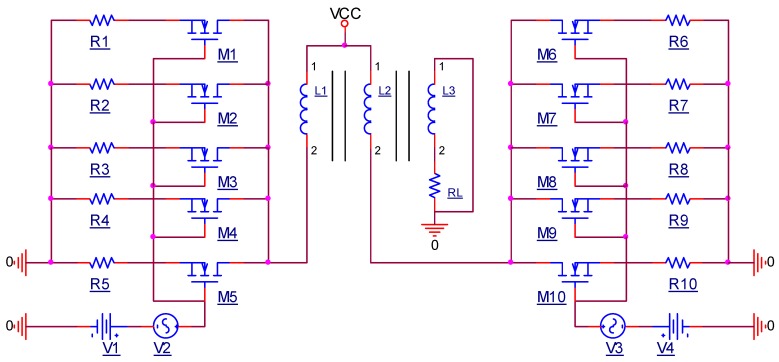
Linear amplification parallel output circuit with five linear amplification modules (LAMs).

Ideally, when multiple LAMs with identical MOSFETs are set at the same static operating point and identical small AC signals are separately inputted into each MOSFET, the magnified AC current by each MOSFET linear amplifier circuit has identical magnitude and direction for identical load. If the output terminals of each MOSFET LAM are connected in parallel to one load, the amplified AC currents by each MOSFET LAM simultaneously pass through the load, and the current and output power on the load can be increased. Although the output power of the linear PAs in class AB designed with two active elements is very small [23,24,25], when each branch is composed of multiple MOSFET LAMs, whose output terminals are connected in parallel to one end of the power transformer, the output current and output power of the transformer output terminal can be significantly increased. Based on the AB class with transformer output configuration, each branch was designed with five linear LAMs in parallel as the output stage, as is shown in Figure 1. Considering the performance and price, an N-channel power MOSFET IRF840 is selected as the active element of each LAM.

**Figure 2:**
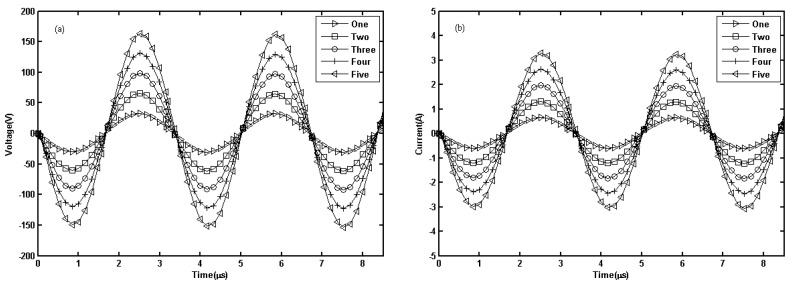
Simulation result of the linear PA with different numbers of LAMs: (**a**) voltage; (**b**) current.

To compare the parallel output performance of different numbers of LAMs, the PSpice simulation of a linear PA consisting of one, two, three, four, and five MOSFETs is performed. The spice models of the key component (IRF840) were provided by the vendors. The simulation result is displayed in Figure 2. The voltage amplitudes of one, two, three, four, and five LAMs loaded on 50 Ω are 62.91 V, 125.93 V, 188.79 V, 251.3 V, and 313.2 V, respectively. The current amplitudes of one, two, three, four, and five LAMs are 1.2583 A, 2.519 A, 3.776 A, 5.025 A, and 6.264 A, respectively. The results show that the voltage or current amplitudes on the load are approximately proportional to the number of parallel LAMs. The output peak powers of one, two, three, four, and five LAMs are 79.16 W, 317.22 W, 712.87 W, 1262.78 W, and 1961.88 W, respectively. The results show that the peak output power increases approximately with the square of the number of parallel LAMs. However, there are more parallel LAMs in the actual circuit; the stronger driving ability of the required front circuit corresponds to greater inter stage crosstalk and lower stability of the circuit. Therefore, it is necessary to select the maximum number of parallel LAMs according to the actual debugging results.

### 2.2. Gated Linear PA Configuration

**Figure 3:**
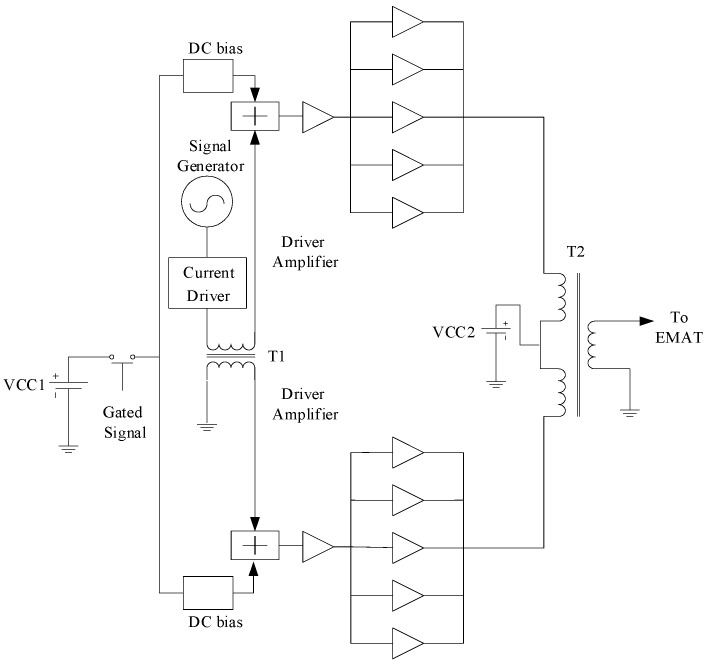
Gate configuration of the proposed linear PA.

The linear PA functions for a very short time (such as tens of µs) and rests for a very long time (such as from tens to hundreds of ms) in one detection process, so the continuous linear PA is not suitable for ultrasonic guided wave inspection. The gated mode can be used to run the linear PA in a linear amplification mode, where the burst signal is instantaneously transmitted; then, the linear PA is stopped during the resting period of the detection process. Therefore, the power consumption of the proposed linear PA can be significantly reduced. However, the gated mode will often turn on and off the linear PA, which causes the inevitable disruption in the output stage in the range of tens of volts because of the switching characteristics of the circuits. If these disruptions are delivered to the EMAT, they cause inherent noise in the time history curve, and it is easy to induce the fault decision. Therefore, suppressing the disruptions in the output stage caused by the switching characteristics is a key problem that must be carefully considered in the development of the linear PA. In addition, AB class with Transformer output must input the differential AC signals. To resolve these problems, the proposed linear PA for EMAT excitation was designed with the configuration in Figure 3.

A single-to-differential conversion circuit with transformer T1 was adopted in the input stage of the linear PA. Each branch of the differential structure consists of five LAMs with output terminals that are connected in parallel to increase the output power. Five LAMs are driven by one driver amplifier. Each LAM has negative voltage feedback to extend the bandwidth. A differential to single-ended conversion circuit with transformer T2 was adopted in the output stage. A gated signal that is synchronous with the arbitrary waveform burst signal emitted from the signal generator is used to switch on the power to the DC bias circuit. The DC bias circuit is used to set a quiescent operation point for each branch of the differential structure. The voltage and current of the arbitrary waveform burst signal emitted from the signal generator are amplified by the current driver to improve the driver capability of the arbitrary waveform burst signal.

When the gate is opened, the inputted burst signal is converted into two equal-amplitude burst signals with a phase difference of 180° by T1. Then, two burst signals of equal amplitude with a phase difference of 180° are each amplified by one of two identical branches, and the amplified high-power signals also have equal amplitudes and a phase difference of 180°. Two amplified high-power signals are combined with transformer T2 and deliver the total power to the EMAT. Because each branch of the differential circuit is theoretically identical, the disruption in the output stage of each branch caused by circuit-switching characteristics should also be identical when the same gate signal is inputted to each branch. Therefore, this differential structure should suppress the disruption that would occur at the output stage of each branch.

## 3. Implementation

### 3.1. AC and DC Coupling Circuit

**Figure 4:**
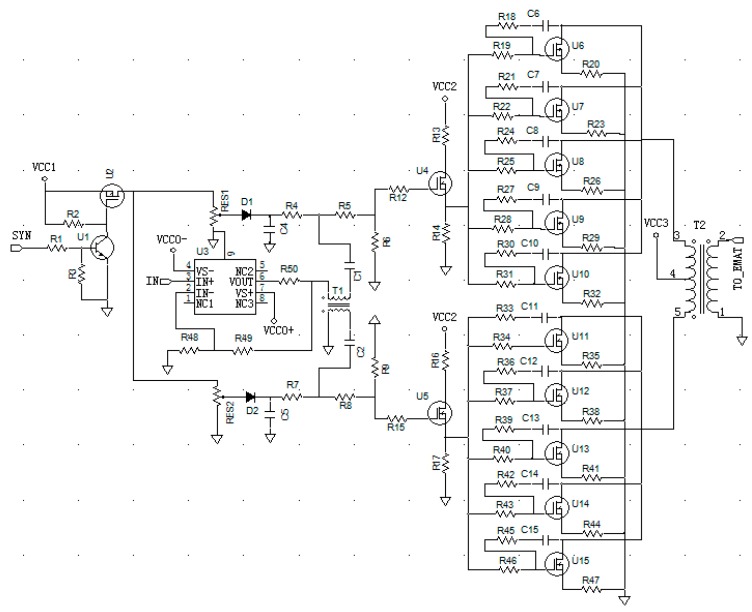
Proposed linear PA including two driver amplifier and ten power amplifier modules.

The AC and DC coupling circuits are shown in Figure 4. When the level of the gated signal inputted from the SYN port is low, the DC power supply VCC1 cannot power the DC bias circuit and the power amplifier circuit is expected to stop working. When the level of the gated signal inputted from the SYN port is high, the collector and emitter of the n-p-n amplifier transistor U1 (2N5551, Motorola, Chicago, IL, USA) are in a conducting state. The gate pole of the p-channel power MOSFET U2 (IRF9540, Vishay, Malvern, PA, USA) is connected with the ground plane through a conducting resistor between the collector and the emitter of the amplifier transistor U1. Therefore, the p-channel power MOSFET U2, which has a drain-source on-state resistance of less than 0.2 Ω, is a conducting element. One electrical terminal that consists of two sliding rheostats is connected to the power supply VCC1 through the drain-source on-state resistance of U2, and the sliding contact of the two rheostats is used to set up the appropriate biasing voltage for the driver amplifier circuits. Because the components of the two branches cannot be exactly identical, a small amplitude disruption in the output stage may always be present. This small amplitude disruption can be suppressed by finely tuning the two sliding rheostats.

The arbitrary waveform burst pulse inputted from the IN port is amplified by high-voltage, high-speed, current-feedback amplifier U3 (THS3091, Texas Instruments, Dallas, TX, USA). The high slew rate of over 7300 V/s and large output current of over ±250 mA of the THS3091 make it sufficient to drive two driver amplifiers. The wideband transmission line transformer T1, which is fabricated from two strands of enamel-coated copper wire twisted together and wound around a wideband ferrite core 20 times, is used to convert the single-ended signal outputted from U3 into differential signals. The wideband ferrite core of T1 is made from a soft ferrite magnet (MXO-2000, Flight Bo De Electronic component, Beijing, China) with an inner diameter of 10 mm, an outer diameter of 16 mm, and a height of 5 mm. Two differential burst signals are added into the gated DC biasing signals with capacitors C1 and C2. The AC coupling mode eliminates the effects of DC signals on the signal generator. The main function of the diode (1N4148, Chenyi Electronics, Shanghai, China) is to prevent the AC signal interference with the power supply. Because the driver amplifier produces the load for the AC and DC coupling circuits, the rise and fall time of the gate DC bias signal should be regulated to reduce the power consumption and associated heat from the opening of the linear PA. Capacitors C4 and C5 can be selected to reduce the rise time of the DC bias voltage and filter the AC signal leaked to the power source. Resistors R6 and R9, which are parallel in the input port of the driver amplifier circuit, can be used to regulate the fall time of the DC bias voltage. When the SYN port has been triggered by a task transaction level (TTL) pulse outputted from the signal generator, the linear PA is functional after a rising time caused by the AC and DC coupling circuit. Therefore, the trigger pulsing time for the signal generator should be delayed by this time duration.

### 3.2. Amplifier Module

One branch of the power amplifier includes one driver amplifier and five LAMs. One n-channel power MOSFET (IRF520, Intersil, Milpitas, CA, USA) is the active component in each driver amplifier. The source poles of the two IRF520s are used as the driving ports. One n-type RF MOSFET (IRF840, Intersil) is the active component in each LAM. Each of the two source poles of the two power IRF520s drives five LAMs. Five resistors between the source pole of IRF520 and the gate poles of the five IRF840s prevent mutual interferences between the channels. If the crosstalk between channels can be suppressed, these five resistors should be minimized to reduce the attenuation of the burst signals that run through them. A negative feedback loop is placed between the drain and the gate of each IRF840 to extend the bandwidths of each LAM, so the bandwidth of the linear PA can be correspondingly extended.

The signals, including both gated DC biasing and arbitrary waveform burst signals, are delivered into the gate pole of IRF520. The gated DC biasing voltage in each IRF520 gate pole sets the appropriate quiescent operation point for the two driver amplifier modules, which simultaneously amplify the arbitrary waveform burst signals. The gated DC biasing voltage in each power IRF520 source pole adjusts the appropriate quiescent operation point for five LAMs. The drains of the five IRF840s are parallel and connected with transformer T2. T2 was fabricated from seven rings of two strands of enamel-coated copper wire twisted together and seven rings of one strand of enamel-coated copper wire wound around three wideband ferrite cores, which were fixed together with a strong mixture of glue and water. Each wideband ferrite core was made from the soft ferrite magnet (MXO-2000, Flight BoDe Electronic component), with an inner diameter of 20 mm, an outer diameter of 32 mm, and a height of 5 mm. T2 has to two roles: To protect the IRF840s by intercepting abrupt changes in the signal from the DC power supply VCC2 and to implement the differential to single-ended conversion and combine the total power provided by all power amplifier modules, which is delivered to the EMAT.

## 4. Performance Evaluation

**Figure 5:**
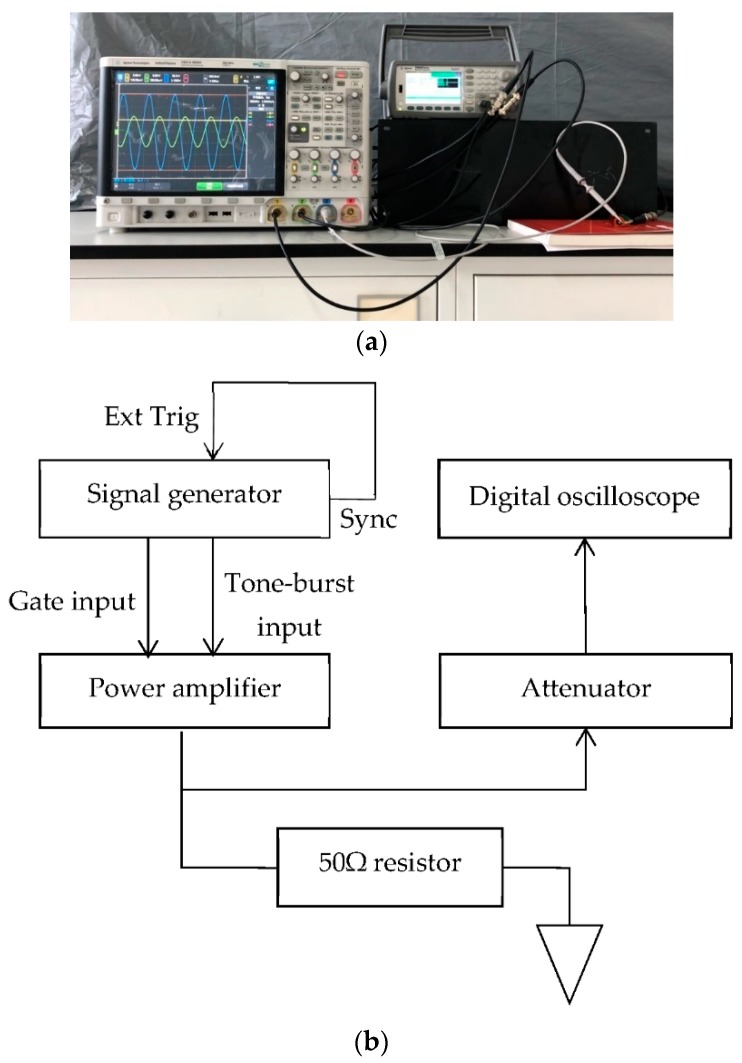
(**a**) Photo and (**b**) schematic of the measurement system to evaluate the performance of the proposed linear PA.

Figure 5 shows a schematic diagram of the measurement system to evaluate the performance of the proposed linear PA. A signal generator (33522B, Agilent, Santa Clara, CA, USA) produced the gated input trigger signal from its output channel 1 and the burst input signal from its output channel 2 to the linear PA. To synchronize the two output channels of the signal generator, it is necessary to connect the Sync output port with the Ext Trig input port and to set the source of output channel 2 as the external excitation mode. One output signal from the linear PA was delivered to a 50 Ω resistor load that can accommodate 10 W of continuous power. The other output of the linear PA was connected to the 10 dB attenuator of a voltage probe (N2894A, Agilent) and delivered to an oscilloscope (DSO-X 4034A, Agilent). The performance of the linear PA was evaluated by recording the voltage across the 50-Ω resistor load. The duration of the gated trigger signal ranged from 15 to 1000 µs, and its pulse repetition rate ranged from 1 to 40 Hz. The frequency of the tone of the source burst signal varied from 0.1 Hz to 30 MHz, and its Vpp voltage changed from 1 mV to 10 V. The delay time of the source burst signal after the gated trigger signal was set as 7 µs by setting up the trigger setup parameter of output channel 2. The oscilloscope digitized the output signal of the attenuator, which was sampled at a rate of 5 GSa/s.

### 4.1. Gating Mode Test

**Figure 6:**
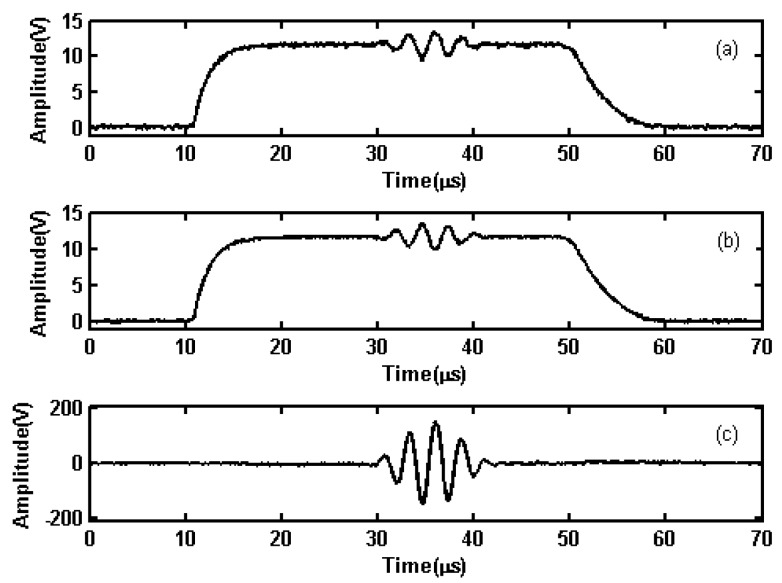
The driving signals of each branch and output signals of the power amplifier: (**a**,**b**) driving waveforms of two branches; (**c**) output waveforms of the output stage.

Gating mode test shows that the power amplifier can work in gating mode and eliminate the disruptions caused by switching characteristics. The high-level width of the gate-controlled signal for powering the DC bias circuit was set to 40 µs. A 5-cycle sinusoidal wave modulated by a Hanning window with a frequency of 360 kHz was inputted into the LPA. Moreover, the amplified signal with 300 V was transmitted at 50 ms interval. The oscilloscope was used to observe the output waveform of AC and DC coupling circuit of each branch, as shown in Figure 6a,b, and the output waveform on 50-Ω resistor load, as shown in Figure 6c. From Figure 6a,b, it is known that the gated signals provided to the two branches rise and fall with 7 µs delay. The delay time should be considered for the AC signal. From Figure 6c, it is known that the output signals are located near the zero line when the whole circuit is turned on or off. This shows that the linear PA not only realizes the gating mode but also can effectively suppress the disruptions caused by the switching characteristics.

### 4.2. Measurement with Typical Guided Wave Source Signals

**Figure 7:**
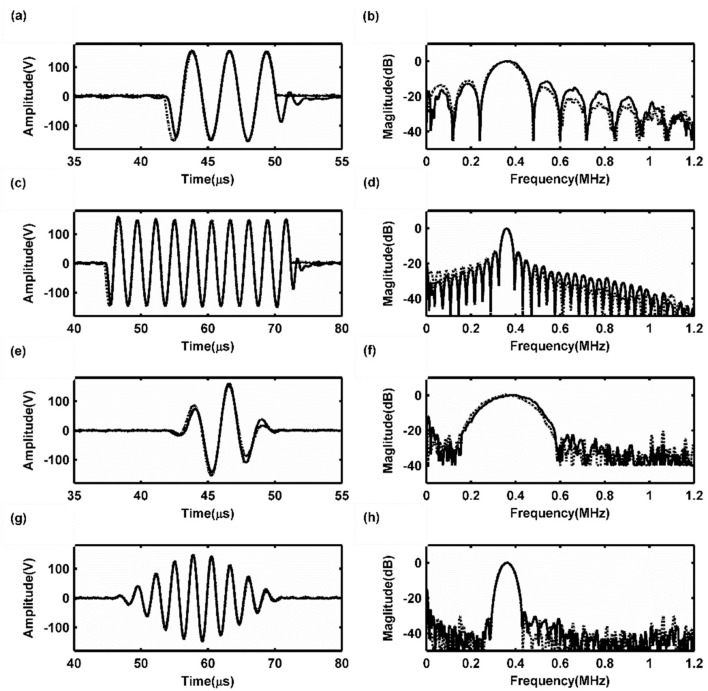
Output results of typical signals: (**a**) a 360-kHz three-cycle sine burst and its spectrum (**b**); (**c**) a 360-kHz ten-cycle sine burst and its spectrum (**d**); (**e**) a Hanning window function-modulated 360-kHz three-cycle sinusoidal signal and its spectrum (**f**); (**g**) a Hanning-window-function-modulated 360-kHz ten-cycle sinusoidal signal and its spectrum (**h**). Solid lines are measured values and dashed lines are ideal waveforms.

Usually, two kinds of signals are employed as guided wave driving source signals: A few cycles of sine signals and Hanning window function-modulated sine signals. In this study both types of source signals were used to test the flexibility of the proposed linear PA. Figure 7 shows the waveforms of three- and ten-cycle sine signals with frequencies of 360 kHz and Hanning windowed three- and ten-cycle sinusoidal signals with center frequencies of 360 kHz. The Vpp of the high voltage waveforms emitted from the linear PA was 300 V. To evaluate the performance of the waveforms emitted by the linear PA, the time domain waveform shape and spectral characteristics of high voltage burst signals and the ideal waveforms for the guided wave application were compared with each other, as shown in Figure 7.

For driving source signals using a few cycles of sine signals, Figure 7a,c show that their waveforms outputted from the linear PA are approximately the same as the ideal waveforms. However, there is a small amount of hysteresis in the first quarter cycle. This is primarily because of the linear PA’s transient response effect. An amplified signal usually requires a transition time to achieve the steady-state response. Therefore, the figure shows that the amplified signal has a slight lag relative to the ideal signal during the transition time. In addition, the amplitude of the amplified signal is slightly smaller than ideal signal during the transition time, and furthermore, a tailing phenomenon appears at the end of the output waveforms. Figure 7b,d shows that the main lobes of the output sine waveform spectra fit well with those of the ideal waveforms; however, the side lobes of the output sine waveform spectra have smaller amplitudes at the lower frequencies and larger amplitudes at higher frequencies. For driving source signals using a few cycles of Hanning windowed sine signals, Figure 7e,g show that the output waveforms fit well with the ideal waveforms and that when the cycle number increases the output and ideal waveforms are almost the same. It should be noted that the transient response effect for Hanning windowed sine signal is smaller than that found for rectangular window sine waves, especially when the modulation cycle is higher. This is primarily because the Vpp of each cycle of Hanning windowed sine signal increases gradually to the maximum value and then decreases to the minimum value. When the modulation cycle number is higher, the amplitude of the first cycle will become small. Therefore, the transient response effect becomes weaker than in cases with fewer modulation cycles. Figure 7f,h show that within the entire frequency domain, the second harmonics of the output waveforms are always smaller than those of the ideal waveforms. Therefore, even the harmonics component of the output waveforms could be suppressed by the differential structures. Both of these two kinds of signals are able to yield the largest peak-to-peak amplitudes and be adopted as the guided wave inspection signals.

### 4.3. Evaluation of Voltage Gain and P1dB Compression Point

When the MOSFETs and output power transformer of the final amplifier stages are not changed, the bandwidth of each LAM can be expanded by adding feedback circuit, and the bandwidth of the linear PA can be correspondingly expanded. The linear PA with and without feedback are measured separately to study the effect of feedback circuits on bandwidth. The output responses of the linear PA were measured by loading 25 cycles of long-duration sine bursts into the signal generator. The amplitudes of waveforms outputted from the linear PA were recorded by the oscilloscope, and then the peak output power was acquired according to the relation

(1) $$P_{\mathrm{out}}=V_{out}^{2}/R_{\mathrm{Load}}$$

In Equation (1), $P_{out}$ is the peak output power for the resistor load, $V_{out}$ is the peak-peak voltage of the resistor load, $R_{Load}$ is the value of the resistor load.

**Figure 8:**
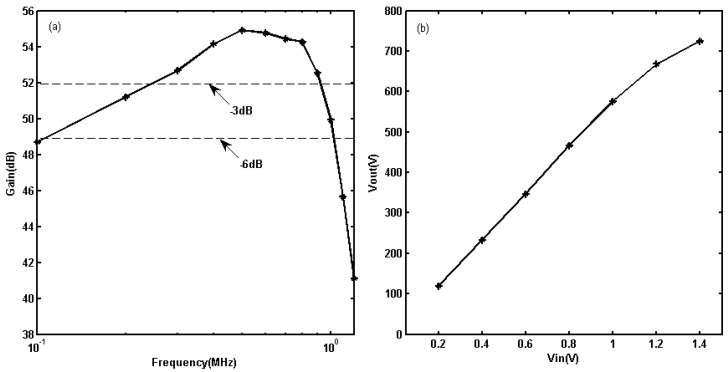
Evaluation of the proposed linear PA without feedback: (**a**) gain distribution from 100 kHz to 1.2 MHz; (**b**) Output voltages with increased input voltages at 500 kHz.

For the voltage gain evaluation of the linear PA without feedback, the frequency of input sine bursts was varied from 100 kHz to 1.2 MHz and amplitudes of input signals were 1.2 Vpp at each frequency variation. The linear PA’s voltage gain curve is shown in Figure 8a. According to Figure 8a, the maximal output power reaches 8.92 kW at 500 kHz. The output powers decrease monotonically in the directions of both the highest and lowest frequencies. The −3 dB point of the low frequency range is 4.46 kW at 243.3 kHz. The −3 dB point of the high frequency range is 4.462 kW at 930 kHz. The center frequency of the linear PA is at approximately 500 kHz, with a −3 dB bandwidth from 243.3 kHz to 930 kHz and −6 dB bandwidth from 106 kHz to 1.03 MHz.

For evaluations of the linear dynamic range of linear PA without feedback, P1dB compression point was measured at the center frequency 500 kHz. Twenty-five cycles of long-duration sine bursts were used to drive the linear PA, and the voltage of input sine bursts was varied from 0.2 to 1.4 Vpp. Figure 8b shows the variation in the output voltages of linear PA for the different input voltages. These results show that the output voltages increase approximately linearly with increasing input voltages when the Vpp of input sine bursts is less than 1.2 Vpp. The output power at P1dB compression point is 9.97 kW.

**Figure 9:**
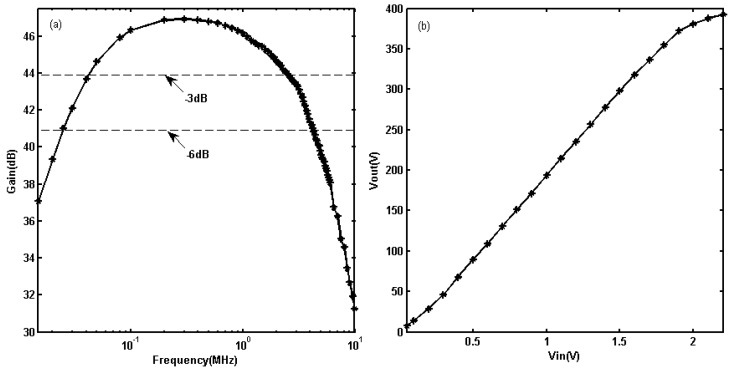
Evaluation of the proposed linear PA with feedback: (**a**) gain distribution from 15 kHz to 10 MHz; (**b**) Output voltages with increased input voltages at 300 kHz.

For the voltage gain evaluation of linear PA with feedback, the frequency of input sine bursts was varied from 15 kHz to 10 MHz, and amplitudes of input signals were 1.8 Vpp at each frequency variation. The linear PA’s voltage gain curve is shown in Figure 9a. According to Figure 9a, the maximal output power reaches 2.52 kW at 300 kHz. The output powers decrease monotonically in the directions of both the highest and lowest frequencies. The −3 dB point of the low frequency range is 1.26 kW at 40 kHz. The −3 dB point of the high frequency range is 1.26 kW at 2.5 MHz. For input sine burst amplitudes of 1.8 V, the output powers are all greater than 1 kW. The center frequency of the linear PA is at approximately 300 kHz with a −3 dB bandwidth from 40 kHz to 2.5 MHz and −6 dB bandwidth from 24.5 kHz to 4.18 MHz.

For evaluations of the linear dynamic range of linear PA with feedback, P1dB compression point was measured at the center frequency 300 kHz. Twenty-five cycles of long-duration sine bursts were used to drive the linear PA, and the voltage of input sine bursts was varied from 0.05 to 2.2 Vpp. Figure 9b shows the variation in output voltages of linear PA for the different input voltages. These results show that the output voltages increase approximately linearly with increasing input voltages when the Vpp of input sine bursts is less than 1.9 Vpp. The output power at P1dB compression point is 2.9 kW.

When each LPA without and with negative voltage feedback, the maximum transient output power reduces from 8.92 kW to 2.52 kW, but the low and high frequency performance of linear PA is improved. The −3 dB bandwidth has expanded from 930 kHz to 2.5 MHz at high frequency bandwidth. The output power of linear PA without feedback is only 462 W at 1.2 MHz, and the output power of linear PA with feedback is increased to 1.93 kW at the same frequency. The −3 dB bandwidth has expanded from 243.3 kHz to 40 kHz at low frequency bandwidth. The output power of linear PA without feedback is only 1.95 kW at 100 kHz, and the output power of linear PA with feedback is increased to 2.2 kW at the same frequency. Furthermore, at the frequency point of 40 kHz, the output power of the linear PA with feedback circuit is 1.26 kW, but the output waveform of the linear PA without feedback circuit has been seriously distorted. Therefore, the linear PA with feedback circuit is more suitable for EMATs UGW applications.

### 4.4. Evaluation of Different Resistance Effects

**Figure 10:**
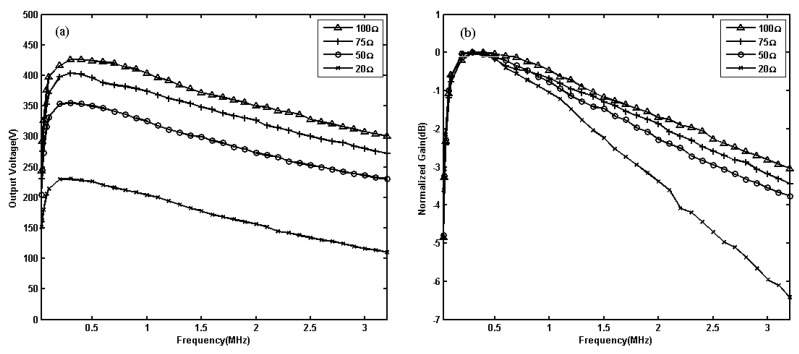
Output voltage (**a**) and normalized gain (**b**) measured under different loads of 20 Ω, 50 Ω, 75 Ω and 100 Ω.

Different material detected and lift-off variation lead to the change of coil impedance in EMAT, which has been confirmed in the research of eddy current in reference [33,34,35,36]. Then, different loads of 20 Ω, 50 Ω, 75 Ω, and 100 Ω were studied. When the input voltage was 1.8 V, the output voltages of different loads were tested in the range of 30 kHz to 3.2 MHz. The results are shown in Figure 10. From Figure 10, we can see that for 20 Ω load, the −3 dB bandwidth is 39 kHz–1.83 MHz, and the maximum voltage gain is 43.16 dB; for 50 Ω load, the −3 dB bandwidth is 40 kHz–2.5 MHz, and the maximum voltage gain is 46.92 dB; for 75 Ω load, the −3 dB bandwidth is 42 kHz–2.9 MHz, and the maximum voltage gain is 48.05 dB; for 100 Ω load, the −3 dB bandwidth is 43 kHz–3.2 MHz, and the maximum voltage gain is 48.51 dB. For the same input voltage, larger load resistance will achieve higher output voltage, larger gain, and wider bandwidth.

From Figure 10, the maximum output power of 20 Ω can reach 2.65 kW; 50 Ω can reach 2.52 kW; 75 Ω can reach 2.18 kW; and 100 Ω can reach 1.82 kW near the central frequency of 300 kHz. In the range below the central frequency, the load is smaller; the output power is larger. However, when the frequency increases to 800 kHz, the output power of 50 Ω is greater than that of 20 Ω. When the frequency increases to 2 MHz, the output power of 100 Ω is greater than that of 20 Ω. This is mainly because the output impedance of the transformer always varies with the frequency, which results in the output impedance of the power amplifier changing with the frequency. In addition, the output characteristics of each amplifier module also vary with frequency.

## 5. EMAT Inspection Experiment and Results

In Section 4, for the convenience of performance evaluation, a 50 Ω resistor was used as a load on the specially designed linear PA. Because EMATs always exhibit complex impedance, the energy transfer process between the output stage of the linear PA and the EMAT is more complicated than when the linear PA excites a resistor load. Therefore, these experiments were required to verify that the linear PA can excite an EMAT to generate ultrasonic guided waves in an actual inspection.

### 5.1. Experimental System

**Figure 11:**
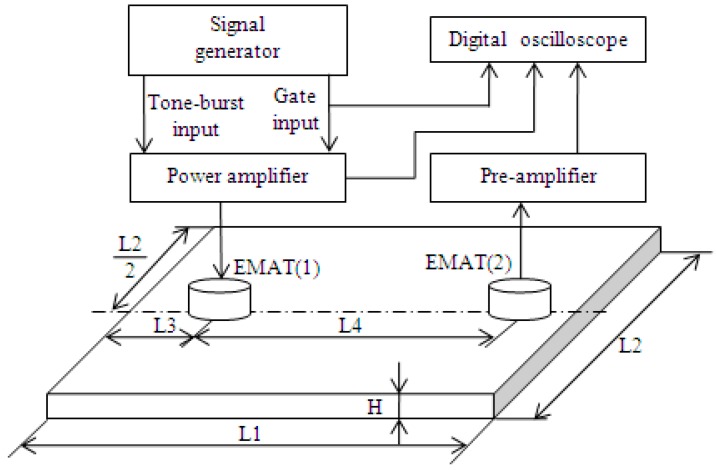
The schematic of electromagnetic acoustic transducer (EMAT)-based guided wave inspection system for the performance evaluation of the linear PA.

Figure 11 shows the experimental system for testing the ability of the linear PA to excite an EMAT for generating ultrasonic guided waves. Pairs of identical EMATs were tested in a pitch-catch configuration on two different plates, and the materials of the plates were listed in Table 1. Planar coil EMATs were developed to excite the single S0 mode according to the principle presented in [2]. The arrangement of two EMATs and the dimensions of the plates used in these experiments were also listed in Table 1.

The gated signal emitted from the synchronic port of the signal generator (33220A, Agilent) was connected to the first channel of a digital storage oscilloscope (TDS2024B, Tektronix, Beaverton, OR, USA). The oscilloscope was triggered at a high TTL by a signal delivered from the signal generator that was longer than the transmitted burst signal over a period of 100 ms. The transmitting EMAT was driven by the output port of the linear PA, which was driven by the signal generator. The signals used to excite the transmitting EMAT were fed into the second channel of the digital storage oscilloscope through a voltage probe (P2220, Tektronix) with the 10 dB attenuator. The receiving EMAT was connected through a specially designed pre-amplifier that provided approximately 92 dB of gain into the third channel of the digital storage oscilloscope. To eliminate random noise, the received signals were averaged 32 times in all experiments.

### 5.2. EMAT Inspection Experiment and Results

To demonstrate the potential of the linear PA for NDT and NDE applications, two types of experiments were designed: (1) to demonstrate the exciting capability of the output signals with different amplitudes and (2) to show the exciting capability of the output signals with different frequencies.

**Figure 12:**
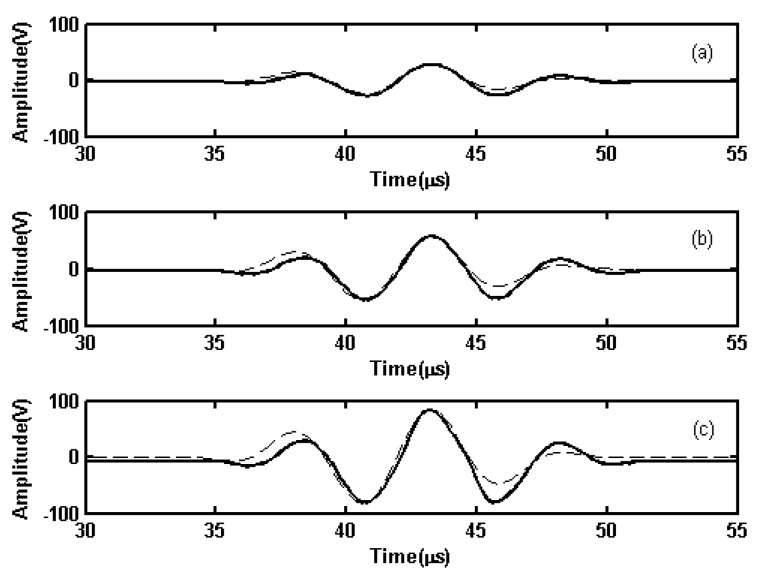
Output results of Hanning-window-function-modulated 180-kHz three-cycle sinusoidal signals with Vpp of (**a**) 56 V, (**b**) 112 V, (**c**) 163 V when the EMAT was used as the load. Solid lines are measured values, and dashed lines are ideal waveforms.

In Experiment 1, contrastive tests were conducted to demonstrate the influence of output voltage amplitudes on the signal-to-noise (S/N) ratio of the received signals. When three Hanning windowed sinusoidal signals with center frequencies of 180 kHz and Vpp of 0.2 V, 0.6 V, and 1 V, respectively, were outputted from the signal generator, amplified signals with Vpp of 56 V, 112 V, and 163 V were outputted from the linear PA to the transmitting EMAT in an Al plate, as shown in Figure 12. These experimental results demonstrate that the output waveforms of different amplitudes from the exciting EMAT all produce the largest amplitudes with the least distortion; however, the distortion at the beginning and end of the waveform is higher than the distortion when the linear PA is used to excite the resistor. The primary reasons for this difference are the resonant nature of the linear PA and the interaction of its output impedance with the impedance of the EMAT. It was challenging to avoid the distortion entirely; however, it had minimal impact on the practical detection results.

**Figure 13:**
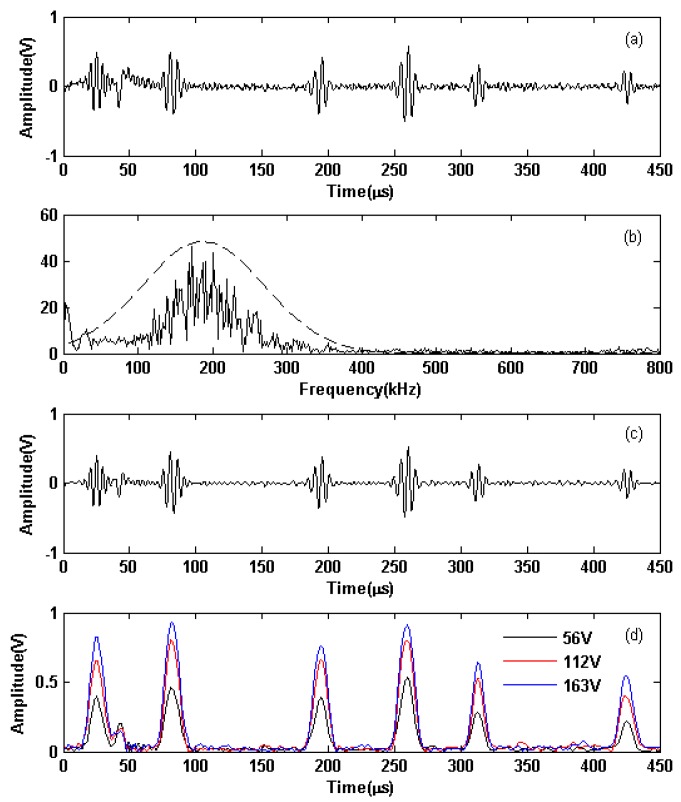
Inspection results in an Al plate when the exciting signals were with center frequencies of 180 kHz: (**a**) received signal when the exciting signals’ Vpp of 56 V; (**b**) spectra of the received signal when the exciting signals’ Vpp of 56 V (solid lines) and the Gaussian function (dashed lines); (**c**) filtered received signal when the exciting signals’ Vpp of 56 V; (**d**) Hilbert envelope of the filtered received signal when the exciting signals’ Vpp of 56 V, 112 V, and 163 V.

The output waveforms from the transmitting EMAT are shown in Figure 12a, and the received signal from the receiving EMAT are shown in Figure 13a. The spectra of the received signal in Figure 13a are shown in Figure 13b, and the filtered received signal are shown in Figure 13c. Hilbert envelopes of the filtered received signals with Figure 12a–c as exciting signals are shown in Figure 13d. In Figure 13d, the first, second, and third wave packet appears at 25.0, 81.6, and 194.4 µs, respectively. The first wave packet is the electromagnetic cross-induced signal from the transmitting EMAT, which can be used as the exciting reference time signal. In accordance with the group velocity of aluminum plate with 1.5 mm thickness, the group velocity of the S0 mode is 5.334 m/ms at 180 kHz. The second wave packet is regarded as the directive waves, and the distance as calculated from the inspection result is 301.9 mm, an error of 0.63% to the actual distance. The third wave packet consists of the reflective echoes from the left end of the plate, and its distance as calculated from the inspection result is 903.6 mm, an error of 0.40% the actual distance.

The S/N ratio was acquired according to the relation [8]:

(2) $$SNR_{\mathrm{dB}}=20\log_{10}(A_{\mathrm{signal}}/A_{noise})$$

In Equation (2), $SNR_{\mathrm{dB}}$ is the signal-to-noise ratio of the signals, $A_{\mathrm{signal}}$ is the maximum amplitude in the second wave, $A_{noise}$ is the average value of noise signal amplitudes between the second and third wave packet.

The S/N ratios of Hilbert envelopes of the filtered received signals in Figure 13d with Figure 12a–c as exciting signals respectively are 12.11 dB, 26.52 dB, 29.19 dB and the amplitudes of the directive waves are 0.46, 0.8, and 0.93 V. From the experimental results, the amplitudes of the exciting signals are larger, and the S/N ratio is better when the other inspection conditions are the same. When the amplitudes of the exciting signals increase, the amplitudes and S/N ratios of the echoes also increase. Therefore, it would be beneficial to improve the S/N ratios to increase the output power of the linear PA.

**Figure 14:**
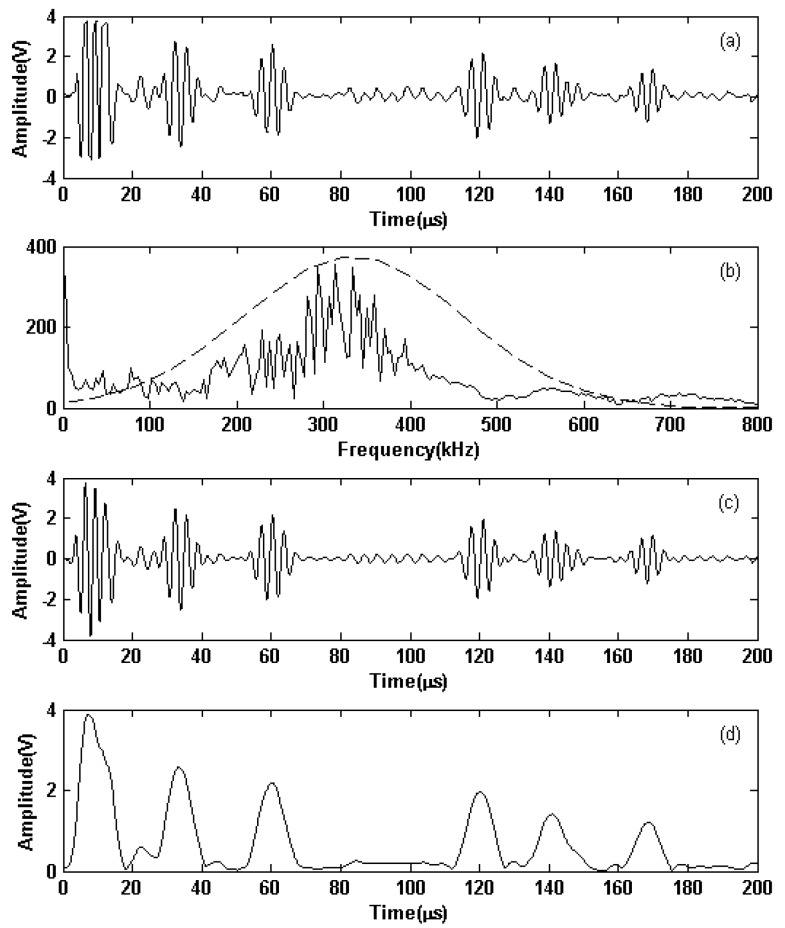
Inspection results in a Ferrum plate when the exciting signals were with center frequencies of 360 kHz and Vpp of 264 V: (**a**) received signals; (**b**) spectra of the received signal (solid lines) and the Gaussian function (dashed lines); (**c**) filtered received signal; and (**d**) Hilbert envelope of the filtered received signal.

To show that the linear PA could excite the EMAT to generate a guided wave in a different frequency, Hanning windowed three-cycle sine bursts at 360 kHz with 236 Vpp outputted from the linear PA were used to excite the EMAT to generate guided waves in a Ferrum plate in Experiment #2. The received signals from the receiving EMAT are shown in Figure 14. In Figure 14d, the first, second, and third wave packet appears at 7.3, 33.4, and 60.2 µs, respectively. The calculated results for the directive waves and the reflective echo from the left end of the plate have an error of 0.41 and 0.93%, respectively, relative to the actual position. The experimental result shows that a high S/N ratio and good detection accuracy are achieved, and verifies the exciting capability of the different frequency signals emitted from the linear PA.

The test results from Experiment 1 and Experiment 2 show that the linear PA can efficiently excite the EMAT to generate ultrasonic guided waves, and the inspected results satisfy the requirements for the application. Therefore, the driving capability of the linear PA is sufficient, not only to drive the resistor load but also to drive the EMAT, and can be effectively used in an electromagnetic ultrasonic guided wave detection system.

## 6. Conclusions

A customized linear PA for electromagnetic ultrasonic guided wave inspection was developed. The amplifier adopts a differential circuit structure to suppress the output disruption and even harmonics waves. The output terminals of many LAMs are connected in parallel to increase the output power. The differential circuit structure doubles the output power to greater than 1 kW in its −3 dB bandwidth. The −3 dB bandwidth of the linear PA is broadened from 40 kHz to 2.5 MHz by negative voltage feedback. The testing results show that the linear PA can be used for exciting EMATs to generate guided waves in a metallic plate structure, and has excellent potential to be used as general equipment for exciting EMATs because of its high instantaneous power output, good linearity, and broad bandwidth.

## Figures and Tables

**Table 1:** Geometric parameters and materials in experiments.

Experimental No.	Materials	L1/mm	L2/mm	H/mm	L3/mm	L4/mm
1	Al	1200	1200	3	300	300
2	Fe	500	600	1.5	70	230
